# Evaluation of the Diagnostic Efficacy of the AI-Based Software INF-M01 in Detecting Suspicious Areas of Bladder Cancer Using Cystoscopy Images

**DOI:** 10.3390/jcm13237110

**Published:** 2024-11-24

**Authors:** Jongchan Kim, Won Sik Ham, Kyo Chul Koo, Jongsoo Lee, Hyun Kyu Ahn, Jae Yong Jeong, Sang Yeop Baek, Su Jin Lee, Kwang Suk Lee

**Affiliations:** 1 Department of Urology, Urological Science Institute, Yonsei University College of Medicine, Seoul 03722, Republic of Korea; lumpakcef@yuhs.ac (J.K.); uroham@yuhs.ac (W.S.H.); js1129@yuhs.ac (J.L.); 2 Department of Urology, Yongin Severance Hospital, Yonsei University Health System, Yongin 16995, Republic of Korea; 3 Department of Urology, Gangnam Severance Hospital, Yonsei University College of Medicine, Seoul 06273, Republic of Korea; gckoo@yuhs.ac; 4 Department of Urology, Ewha Womans University Seoul Hospital, Seoul 07804, Republic of Korea; wharang11co@gmail.com; 5 Department of Urology, National Health Insurance Service Ilsan Hospital, Goyang 10444, Republic of Korea; urojjy@nhimc.or.kr; 6 Infinyx Corporation, Daegu 42988, Republic of Korea; tkdduq631@gmail.com (S.Y.B.); leesjin1219@gmail.com (S.J.L.)

**Keywords:** artificial intelligence, cystoscopy, bladder cancer

## Abstract

**Background/Objectives:** We aimed to evaluate the accuracy of the artificial intelligence (AI)-based software INF-M01 in diagnosing suspected bladder tumors using cystoscopy images. Additionally, we aimed to assess the ability of INF-M01 to distinguish and mark suspected bladder cancer using whole cystoscopy images. **Methods**: A randomized retrospective clinical trial was conducted using a total of 5670 cystoscopic images provided by three institutions, comprising 1890 images each (486 bladder cancer images and 1404 normal images). The images were randomly distributed into five sets (A–E), each containing 1890 photographs. INF-M01 analyzed the images in set A to evaluate sensitivity, specificity, and accuracy. Sets B to E were analyzed by INF-M01 and four urologists, who marked the suspected bladder tumors. The Dice coefficient was used to compare the ability to differentiate bladder tumors. **Results**: For set A, the sensitivity, specificity, accuracy, and 95% confidence intervals were 0.973 (0.955–0.984), 0.921 (0.906–0.934), and 0.934 (0.922–0.945), respectively. The mean value of the Dice coefficient of AI was 0.889 (0.873–0.927), while that of clinicians was 0.941 (0.903–0.963), indicating that AI showed a reliable ability to distinguish bladder tumors from normal bladder tissue. AI demonstrated a sensitivity similar to that of urologists (0.971 (0.971–0.983) vs. 0.921 (0.777–0.995)), but a lower specificity (0.920 (0.882–0.962) vs. 0.991 (0.984–0.996)) compared to the urologists. **Conclusions**: INF-M01 demonstrated satisfactory accuracy in the diagnosis of bladder tumors. Additionally, it displayed an ability to distinguish and mark tumor regions from normal bladder tissue, similar to that of urologists. These results suggest that AI has promising diagnostic capabilities and clinical utility for urologists.

## 1. Introduction

Bladder cancer (BCa) is the 10th most common malignancy worldwide [1], presenting significant challenges in diagnosis and treatment due to its high recurrence rate and the need for ongoing monitoring. White-light cystoscopy (WLC) is the gold standard method for BCa diagnosis and surveillance. Patients with suspicious lesions identified via WLC typically undergo transurethral resection of bladder tumors (TURBT) for definitive pathological diagnosis and staging. Non-muscle-invasive BCa, which comprises approximately 75% of all cases, can often be managed with TURBT [2,3], followed by regular cystoscopic surveillance every 3–6 months to detect recurrence [4].

Despite its widespread use, WLC has several limitations. It is particularly prone to missing flat lesions, such as carcinoma in situ (CIS) and very small tumors, resulting in a misdiagnosis rate as high as 20%–30% [5] and incomplete resection rates of up to 50% [6]. These diagnostic shortcomings contribute to high rates of early recurrence and progression in patients with BCa. Enhancements to cystoscopy, such as narrow-band imaging and photodynamic diagnosis, have been developed to address these limitations, but their adoption has been limited due to the need for specialized equipment and additional training [5].

In recent years, artificial intelligence (AI) has emerged as a transformative technology in medical diagnostics, offering the ability to extract and analyze complex imaging data automatically. AI applications have shown promising results across various fields, including radiology, dermatology, and gastroenterology, by enhancing diagnostic accuracy and efficiency [7,8,9]. AI has also been studied in the field of bladder cancer diagnosis, showing a favorable capacity to detect, stage, or grade bladder cancer based on computed tomography (CT), magnetic resonance imaging (MRI), Hematoxylin and Eosin staining, and urine cytology images [10,11,12,13]. Numerous studies have been conducted on the ability of AI to diagnose BCa based on cystoscopic images accurately. Most of these studies demonstrate a commendable level of accuracy [14,15,16,17,18,19,20,21,22,23].

In keeping with this trend, based on a previous study, we developed AI-based software called INF-M01, version 1.0.0, that automatically marks the boundaries of suspicious areas of bladder cancer in cystoscopy images. In this clinical trial, we aimed to investigate the performance of INF-M01, which automatically analyzes cystoscopic images and detects areas suspected of bladder cancer.

## 2. Materials and Methods

### 2.1. Sample Selection Criteria

This study was designed as a randomized, retrospective, confirmatory clinical trial. We reviewed cystoscopic images of patients who underwent cystoscopy to differentiate bladder tumors from other suspected bladder conditions. The inclusion criteria for the normal group were as follows: (1) male patients undergoing cystoscopy to evaluate their treatment response to benign prostatic hyperplasia, (2) female patients undergoing cystoscopy due to urinary symptoms, and (3) patients undergoing cystoscopy to differentiate microscopic hematuria. None of the patients in the control group were diagnosed with bladder tumors. The inclusion criteria for the cancer group were as follows: (1) patients with suspected bladder tumors on imaging (ultrasound/CT/MRI) confirmed to have bladder cancer through biopsy after cystoscopy and (2) patients previously diagnosed with bladder cancer who underwent cystoscopy for follow-up and were confirmed to have bladder cancer through biopsy.

Exclusion criteria were established to ensure sample integrity. The general exclusion criteria for the cancer group included the following: (1) patients with other cancers, such as rectal or cervical cancer involving the bladder; (2) pregnant women; (3) those whose bladder imaging was obtained using equipment other than the designated endoscopic device; and (4) patients who had not received a histopathological diagnosis through the transurethral resection of bladder tumors. The specific exclusion criteria for the normal group included patients with benign prostatic hyperplasia showing abnormal findings during cystoscopy, those with acute cystitis, those with malignancies in the urinary tract observed on CT or MRI, and those with presumed bladder deformation due to pelvic radiation therapy.

### 2.2. Sample Size Calculation

We initially set the estimated sensitivity and specificity based on previously published studies and the preliminary results from this study to calculate the sample size. The weighted average sensitivity from these two studies was 93.6%, with a lower bound of 90% for the 95% confidence interval. The weighted average specificity was 91.6%, with a lower bound of 89.2% for the 95% confidence interval. Therefore, in this study, we set the estimated sensitivity to 93.6%, expecting a performance of at least 90% at the 95% confidence level, and an estimated specificity of 89.2%, expecting a performance of at least 89.2% at the 95% confidence level. Based on these parameters, the calculated sample sizes were 486 images of bladder cancer and 1404 images for normal controls, respectively. (Supplementary Data S1)

### 2.3. Data Collection and Screening

After receiving approval by the review boards of the three institutions, cystoscopy images containing information on the presence of bladder cancer were collected from Yonsei University Severance Hospital (Approval No.: 1-2022-0052), Gangnam Severance Hospital (Approval No.: 3-2023-0112), the Ewha Woman’s University Seoul Hospital (Approval No.: SEUMC 2022-10-017), and the National Health Insurance Service Ilsan Hospital (Approval No.: NJIMC 2022-09-031). The collected data were sent to Gangnam Severance Hospital for screening. The clinician participating in the reference standard development reviewed each image and excluded those with the following issues: (1) images of poor quality that were too blurry or dark to be interpreted and (2) images unrelated to cystoscopy, such as those taken before the scope entered the bladder. After screening, each institution contributed 486 images from patients with cancer and 1404 images from healthy individuals. 

### 2.4. Establishing the Reference Standard

The reference standard was established using the screened images, and three urologists were involved in its construction. Notably, the specialists involved in this process were distinct from those participating in the interpretation of the cystoscopy images. The reference standard construction process involved extracting image frames from DICOM files containing endoscopic images, processing and uploading these images to labeling software, and having urologists review and label the images based on the presence of a tumor. The labeled images were then categorized into folders containing confirmed cancerous and normal images. This process ensures the reliability and accuracy of the reference standards used in this study.

Cystoscopy images were then assigned unique serial numbers, and interpretation results were recorded along with randomly generated numbers using a Python random shuffle. This randomization process was crucial for maintaining the independence and unbiased nature of the sample data, ensuring that the AI-assisted interpretation and clinical experts received the data in a randomized order.

### 2.5. AI and Urologists’ Interpretation of Cystoscopic Images

**Figure 1:**
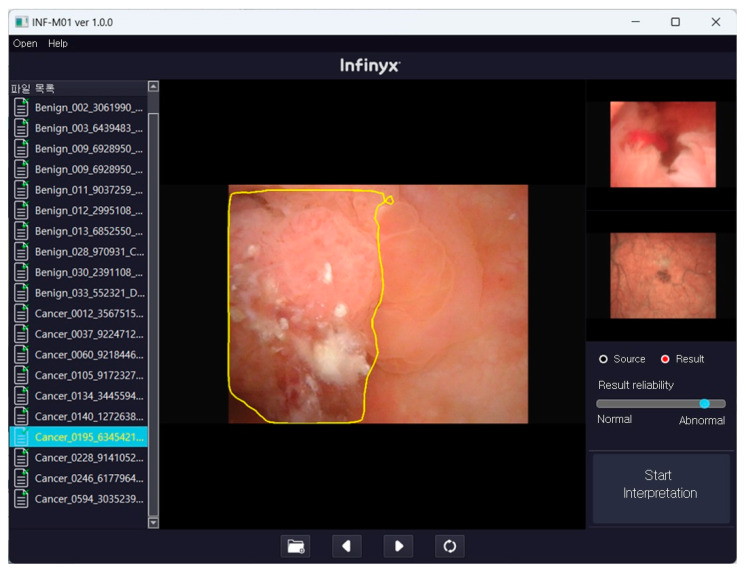
The operating screen of INF-M01. INF-M01 reads the DICOM (Digital Imaging and Communications in Medicine) files in the designated folder and creates a list of cystoscopy images. For each image, the read execution button is clicked to read it, and after the reading is complete, the reading results are displayed on the right. If there is a suspicious area of bladder cancer, the bladder tumor area is marked with a bounding box.

In this clinical trial, five randomly sequenced sets of sample images labeled A, B, C, D, and E were prepared and provided to four clinicians using an AI-assisted diagnostic software for bladder tumors. The analysis was structured as follows: set A was exclusively analyzed using INF-M01, an AI-assisted diagnostic software, with the resulting predicted diagnostic values recorded for each image. The operating screen and reading process of INF-M01 are briefly described in Figure 1. Set A was used to evaluate the performance of AI alone in diagnosing bladder tumors by analyzing the cystoscopic images. In sets B to E, AI and the four urologists (Urologist 1: 12 years of clinical experience, Urologist 2: 17 years of clinical experience, Urologist 3: 9 years of clinical experience, and Urologist 4: 5 years of clinical experience) analyzed the images to determine the presence or absence of suspicious areas of bladder cancer and drew the boundaries of the suspicious areas. Four clinicians independently analyzed this study, each working in separate locations under standard clinical conditions, which included appropriate environments and rest periods to ensure optimal working conditions. The results were reported to the principal investigator after all the analyses were completed. The results interpreted by INF-M01 from sets B to E were compared with those interpreted by the clinicians. The research team at the Gangnam Severance Hospital reviewed and verified the initial results and checked for data manipulation. If no discrepancies were found, the results were forwarded to an external statistical analyst for further analysis.

### 2.6. Statistical Analysis

To evaluate the diagnostic accuracy of AI in interpreting bladder cancer from cystoscopy images, AI interpreted set A 1000 times. Based on these results, the mean values for sensitivity, specificity, accuracy, and the mean Dice coefficient were calculated. The 95% confidence intervals (CIs) were calculated using three different methods: Wald’s continuity correction method, the Jeffreys–Perks method, and Clopper–Pearson’s method.

**Figure 2:**
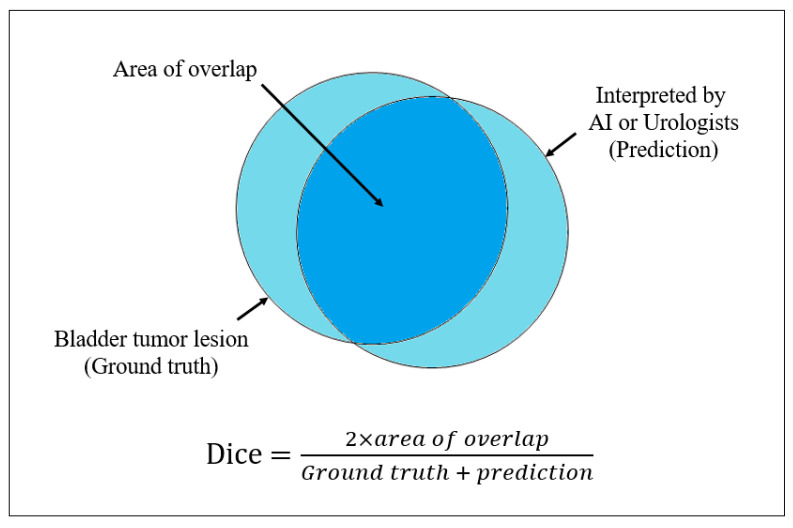
Definition and calculation of Dice coefficient.

Sensitivity, specificity, and accuracy were evaluated for both the AI-assisted diagnostic device and urologists’ interpretations. Additionally, the Dice coefficient was calculated. The Dice coefficient was determined by comparing the bladder cancer regions identified by the AI-assisted diagnostic device and urologist interpretations with the actual bladder cancer regions. If multiple lesions were present, the entire area of all the lesions was included in the calculation. Consequently, one Dice coefficient was generated per image, with values ranging from 0 to 1 (Figure 2). A Dice coefficient of 1 indicates that the region identified by the urologists or the AI-assisted diagnostic device perfectly matches the ground truth. Consequently, the Dice coefficient quantified the proximity of the AI’s predictions to the actual results.

## 3. Results

The interpretation results of INF-M01 for dataset A are shown in Table 1. Out of the 486 cancer images, 473 were identified as cancerous, and 13 were interpreted as normal. Among the 1404 normal images, 1293 were correctly identified as normal. The sensitivity, specificity, accuracy, and 95% confidence intervals (CIs) calculated using Wald’s method with continuity correction were 0.973 (0.955–0.984), 0.921 (0.906–0.934), and 0.934 (0.922–0.945), respectively. The mean Dice coefficient was 0.903 (range, 0.891–0.914). These results exceed the sensitivity of 93.6% and specificity of 89.2% presented in Section 2.2, indicating that INF-01 has achieved our target accuracy in identifying suspected areas of bladder cancer.

Table 2 presents the interpretation results of the remaining four sets by the four urologists and AI. The mean Dice coefficient of urologists ranged from 0.903 to 0.963, and that of AI ranged from 0.873 to 0.927. The urologists’ sensitivity, specificity, and accuracy ranged from 0.777 to 0.995, 0.984 to 0.996, and 0.931 to 0.991, respectively. The sensitivity of AI ranged from 0.952 to 0.977, the specificity from 0.882 to 0.962, and the accuracy from 0.904 to 0.959. The accuracy and mean Dice coefficients were comparable between the clinicians and AI. These findings indicate that, similar to the urologists, AI can independently identify and interpret suspected areas of bladder cancer in cystoscopy images with a high degree of accuracy.

## 4. Discussion

AI has been extensively researched in the field of medicine. In the area of bladder cancer, initial studies have focused on predicting recurrence or survival using machine learning based on clinicopathological data [10,11,12,13]. Medical image analysis leveraging AI was first attempted in fields such as radiology, dermatology, and gastroenterology [7,8,9]. In particular, AI-assisted diagnostic technologies in chest radiology have been used clinically for several years [24]. With the advancement of image analysis methods, numerous studies have been published on the application of machine learning to cystoscopic images for cancer detection (Table 3).

Eminaga et al. [14] developed five deep convolutional neural network (CNN) models by reproducing 479 images from a digital atlas for cystoscopy, resulting in 18,681 images. In their validation results, the most accurate model had an accuracy of 0.99, demonstrating the potential of deep learning for the diagnostic classification of cystoscopic images. Shkolyar et al. [15] developed a deep learning algorithm called CystoNet, demonstrating a sensitivity of 90.9% and a specificity of 98.6% in detecting bladder cancer from cystoscopy videos. Similarly, Wu et al. [20] reported a sensitivity of 97.5% and a specificity of 98.3% for their AI system, which was validated using a large multicenter dataset. This study addresses some of the limitations of earlier research by including a broader range of tumor stages and testing for AI in a real-world clinical setting.

The integration of AI into the diagnostic process of bladder tumors using cystoscopic images represents a significant advancement in the accuracy and efficiency of tumor detection. Thus, we developed INF-M01, AI-based software designed to diagnose bladder tumors by analyzing cystoscopic images and marking the suspected tumor areas, distinguishing them from the surrounding normal bladder mucosa. This study aimed to assess INF-M01’s diagnostic reliability for bladder tumors in cystoscopic images and its ability to detect areas suspected of bladder cancer. Consequently, the sensitivity of INF-M01 for bladder tumor diagnosis was 0.973, and the specificity was 0.921, indicating a better diagnostic ability than we targeted. When comparing the areas marked as suspicious for bladder cancer by AI and urologists, the mean Dice coefficient was 0.873–0.927 for AI and 0.903–0.963 for urologists, indicating that the tumor detection ability of AI was similar to that of urologists. Based on these results, we believe that our AI-based software can help urologists analyze cystoscopic images in clinical practice.

Compared with previous studies, the INF-M01 device demonstrated a sensitivity range that closely matched the sensitivity observed in these previous studies. This similarity in sensitivity indicates that AI can effectively identify a high proportion of true positives, making it a reliable tool for bladder cancer detection. Our results showed similar or slightly improved outcomes for the mean Dice coefficient compared to previous findings published by Ikeda et al. However, in the results for sets B–E, the specificity of INF-M01 was slightly lower than previously reported (0.882–0.962%). Although this was not the primary endpoint of our study when comparing the interpretation of AI and the urologists, the sensitivity of AI in diagnosing bladder tumors was similar to that of the urologists, but the specificity was lower. When reflecting on the factors contributing to low specificity, it appears that the primary cause may be insufficient machine learning on the normal anatomy of the bladder. In the selection of normal cystoscopy images, efforts were made to include those devoid of abnormal findings; however, due to the inadequacy of AI’s machine learning with respect to normal structures, such as rugae or trabeculation, its interpretation of these anatomical features may not have been accurate. Additionally, it is plausible that the AI algorithm was deliberately calibrated to prioritize higher sensitivity. Consequently, while sensitivity exhibited results comparable to those of urologists, specificity was consequently diminished. Consequently, the diagnostic accuracy of our software needs slight improvement in the future.

Our study has some limitations. First of all, since the primary endpoint of our study was to assess AI’s detection capability and compare its performance with that of urologists, evaluating AI’s contribution to a urologist’s diagnostic ability was challenging. Future studies should investigate how AI affects the detection performance of clinicians to assess this accurately. Another limitation is that the model was designed to detect bladder cancer from acquired images, making real-time clinical application difficult. As mentioned earlier, Chang et al. [23] evaluated the feasibility of integrating AI in real time during clinical cystoscopy and the transurethral resection of bladder tumors (TURBT) using live-streaming videos. Improving our model for the real-time diagnosis of bladder cancer is necessary in future studies. However, our study has the strength of comparing the accuracy of AI and urologists in identifying bladder cancer using cystoscopy images. The results showed that urologists had a higher specificity and similar sensitivity to AI. These results are expected to assist urologists in the interpretation of cystoscopic images.

## 5. Conclusions

INF-M01 demonstrated reliable accuracy in diagnosing bladder tumors from cystoscopic images. Additionally, it demonstrated the ability to distinguish and mark tumor regions from surrounding normal bladder tissue, similar to that of urologists. These results suggest that AI has promising diagnostic capabilities and clinical utility for urologists.

## Figures and Tables

**Table 1:** Results of artificial intelligence interpreting cystoscopy images.

		Interpretation of AI		
		Cancer	Normal	Total
Cancer		479	111	584
Normal		13	1293	1306
Sensitivity	Wald’s continuity correction method	95.5–98.4		
	Jeffreys–Perks method	95.6–98.5		
	Clopper–Pearson’s method	95.5–98.6		
Specificity	Wald’s continuity correction method		90.6–93.4	
	Jeffreys–Perks method		90.6–93.4	
	Clopper–Pearson’s method		90.6–93.5	
Accuracy	Wald’s continuity correction method			92.2–94.5
	Jeffreys–Perks method			92.3–94.5
	Clopper–Pearson’s method			92.2–94.5
Mean Dice coefficient		90.3% (89.1–91.4)		

AI = artificial intelligence.

**Table 2:** Comparison of urologist and AI interpretations in four cystoscopy image datasets.

	Sensitivity	Specificity	Accuracy	Mean Dice Coefficient
Urologist 1	0.956	0.996	0.986	0.954
AI	0.977	0.879	0.904	0.876
Urologist 2	0.995	0.989	0.991	0.963
AI	0.983	0.882	0.908	0.879
Urologist 3	0.956	0.993	0.984	0.943
AI	0.952	0.962	0.959	0.927
Urologist 4	0.777	0.984	0.931	0.903
AI	0.971	0.887	0.908	0.873

AI = artificial intelligence. Urologist 1: 12 years of clinical experience, Urologist 2: 17 years of clinical experience, Urologist 3: 9 years of clinical experience, and Urologist 4: 5 years of clinical experience.

**Table 3:** Previous studies of artificial intelligence were used in studies for the detection of bladder cancer using cystoscopy images.

Authors	Year	AI Algorithm or Model	Performance
Eminata et al. [14]	2018	CNN	Accuracy: 0.99
Shkolyar et al. [15]	2019	CNN	Sensitivity: 0.909 Specificity: 0.955
Ikeda et al. [16]	2020	CNN	Sensitivity: 0.90 Specificity: 0.94 AUC: 0.98
Lorencin et al. [17]	2020	ANN	AUC: 0.99
Yang et al. [18]	2021	CNN	Accuracy: 0.969 Sensitivity: 0.968
Du et al. [19]	2021	CNN	Accuracy: 0.969 Sensitivity 0.968
Wu et al. [20]	2022	CNN	Accuracy: 0.977 Sensitivity: 0.987 Specificity: 0.975
Yoo et al. [21]	2022	SVM	Accuracy: 0.992 Sensitivity: 0.993 Specificity: 0.980
Zhang et al. [22]	2023	U-Net	Dice: 0.83
Chang et al. [23]	2023	CystoNet	For cystoscopy Specificity: 0.988 For TURBT Specificity: 0.954

AI = artificial intelligence; CNN = convolutional neural network; ANN = artificial neural network; SVM= support vector machine.

## Data Availability

The datasets generated and/or analyzed during the current study are not publicly available due to the policy of our institution.

## Supplementary Materials

The following supporting information can be downloaded at: https://www.mdpi.com/article/10.3390/jcm13237110/s1, S1: Sample size calculation.
