# Supplementary material for: Evaluation of the Diagnostic Efficacy of the AI-Based Software INF-M01 in Detecting Suspicious Areas of Bladder Cancer Using Cystoscopy Images
Source: J Clin Med. 2024 Nov 24;13(23):7110. doi: 10.3390/jcm13237110 (PMC11642196; doi:10.3390/jcm13237110)
Supplement: Supplementary file 1 [file jcm-13-07110-s001.zip › jcm-3288405-supplementary.pdf]

Supplementary S1

Sample size calculation

The sample size calculation was based on the formula provided in the In Vitro Diagnostic Medical Device Approval Review Guidelines from the Ministry of Food and Drug Safety.

$$n = \frac{\left(Z_{\frac{\alpha}{2}} + Z_{\beta}\right)^2 P_1(1 - P_1)}{(P_1 - P_0)^2}$$

Here, P_1 represents the estimated sensitivity (or specificity) of the device, P_0 denotes the lower bound of the clinical target sensitivity (or specificity) confidence interval, $Z_{\alpha/2}$ is the critical value for Type 1 error, and Z_{β} is the critical value for Type 2 error.

To achieve a power of 90% at a significance level of 5%, the required number of bladder cancer patient images was calculated as 486, given an estimated sensitivity of P_1=0.936, a 95% confidence interval lower bound of P_0=0.900, a significance level of α=0.05 and a power of $1-\beta$=0.90.

$$n = \frac{\left(Z_{\frac{\alpha}{2}} + Z_{\beta}\right)^2 P_1(1 - P_1)}{(P_1 - P_0)^2} = \frac{(1.96 + 1.28)^2 \times 0.936 \times (1 - 0.936)}{(0.936 - 0.900)^2} \approx 486.$$

For normal controls without bladder cancer, to achieve a power of 90% at a significance level of 5%, the required number of images was calculated as 1,404, given an estimated specificity of P_1=0.916, a 95% confidence interval lower bound of P0=0.892, a significance level of α=0.05, and a power of $1-\beta$=0.90.

$$n = \frac{\left(Z_{\frac{\alpha}{2}} + Z_{\beta}\right)^2 P_1(1 - P_1)}{(P_1 - P_0)^2} = \frac{(1.96 + 1.28)^2 \times 0.916 \times (1 - 0.916)}{(0.916 - 0.892)^2} \approx 1{,}404.$$
